# Cost-effectiveness of adding-on new antiepileptic drugs to conventional regimens in controlling intractable seizures in children

**DOI:** 10.1186/2008-2231-20-17

**Published:** 2012-08-30

**Authors:** Zahra Gharibnaseri, Abbas Kebriaeezadeh, Shekoufeh Nikfar, Gholamreza Zamani, Akbar Abdollahiasl

**Affiliations:** 1 Department of Pharmacoeconomics and Pharmaceutical Administration, Faculty of Pharmacy, Tehran University of Medical Sciences, Tehran, Iran; 2 Department of Toxicology and Pharmacology Faculty of Pharmacy, Tehran University of Medical Sciences, Tehran, Iran; 3 Food and Drug Laboratory Research Center, Ministry of Health and Medical Education, Tehran, Iran; 4 Department of Pediatrics, Faculty of Medicine, Tehran University of Medical Sciences, Tehran, Iran

**Keywords:** Cost-effectiveness, New antiepiletics, Intractable seizures, Children, Incremental cost-effectiveness ratio

## Abstract

**Background and purpose of the study:**

Intractable seizures are a subgroup of epileptic disorders challenging the physicians’ skills to become controlled. Showing resistance towards common pharmacotherapy, they demand newer antiepileptic drugs acquired at higher costs. 0.06% of children around the world are estimated to suffer from epilepsy and its consequences. The aim of the present study has been to evaluate the cost-effectiveness of these drugs in the treatment of intractable seizures in children.

**Methods:**

Clinical and cost data were collected from medical and cost records preserved at a neurologist office and a referral pharmacy respectively. Based on the new AED which are accessible in Iran, regimens were categorized into eight groups. The first group consisting of conventional AEDs was considered as comparator and the effectiveness of other groups was compared with it. Incremental Cost-effectiveness Ratio (ICER) of adding-on each new antiepileptic drug was calculated in terms of Rials per consequence (Rls/consq) and compared with each other. Furthermore ICER of the regimens was compared with the GDP per capita (Gross Domestic Product) of the year (2010).

**Results:**

the ICER of the adding-on regimens range from negative values for Gabapentin, Levetiracetam and Zonisamide to low values for Lamotrigine (~ 6.4 million Rials/consequence [mil Rls/consq]) and Oxcarbazepine (~7.7 mil Rls/consq) and followed by high values for Topiramate (~21 mil Rls/consq) and Vigabatrin (~43.7 mil Rls/consq) considering the three months of remaining on regimen. By increasing the limit of remaining time to six months, the previously mentioned regimens persist on negative values. However Oxcarbazepine (~28.7 mil Rls/consq) and Lamotrigine (~13.8 mil Rls/consq) show a steep increase. Topiramate (~23.6 mil Rls/consq) displays a less change. Opposite to other regimens, the ICER value of Vigabatrin (~17.26 mil Rls/consq) has shown an important increase.

**Major conclusions:**

Adding-on new antiepileptics to conventional regimens are cost-effective and justified considering the GDP per capita.

## Introduction

Epilepsy is defined as a neurological disorder of brain portrayed by persisting predisposition to develop epileptic seizures [1]. A proportion of 6 over 1,000 children around the world are estimated to suffer from epilepsy and its psychological, social and intellectual development consequences [2]. Known as one of the most common neurological disorders worldwide, epilepsy has several treatment options. However pharmacotherapy remains the mainstay [3]. Since 1993 a high increase in emerging antiepileptic drugs (AEDs) has been observed [4]. The Anatomical Therapeutic Chemical (ATC) classification system of World Health Organization (WHO) collaborating center identifies 45 medicinal substances used in the treatment of epilepsy. Among which 13 are approved and prescribed in Iran. A common style of classifying AEDs is based on the year of introduction to the market. In this perspective, AEDs fall into two categories. Conventional AEDs including Carbamazepine, Ethosuximide, Phenytoin, Phenobarbital, Primidone and Valproate and the newer AEDs including Gabapentin, Levetiracetam, Lamotrigine, Oxcarbazepine, Topiramate, Vigabatrin, Zonisamide. It should be noticed that financial supports are devoted to pharmaceuticals comprised in essential drugs list of Iran [5], resulting in the high prescription of them [6]. While the first generation of AEDs prescribed as monotherapy shows promising results in almost 70% of patients with epilepsy, polytherapy or adding-on newer AEDs is demanded by the rest of the epileptic patients whom are believed to meet intractable seizures [7].

In general, selecting an AED is performed on the basis of many factors including relative efficacy, drug-drug interactions, tolerability and cost [8]. The acquisition costs of newer AEDs are generally higher than the older; nevertheless there superiority in controlling seizures has to be established. Dealing with restricted budget, clinicians and decision-makers are interested in Pharmacoeconomics studies, which measure and balance costs and clinical outcomes of alternative medications. In spite of this, there is a tragic lack of literature on this issue in Iran; however a few of them that have been published in recent years could provide valuable information for decision making [9]. Furthermore, owing to the pharmacogenetics effect and the diversity of genotypes, applying effectiveness data across different countries is difficult [10].

The aim of this study was to compare cost-effectiveness of newer AEDs being added to conventional regimens in treating intractable seizures in children.

## Methods

**Figure 1:**
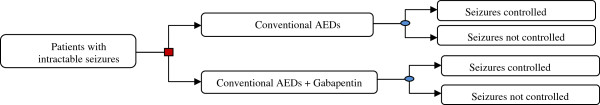
Sample of model used to compare the effectiveness of adding-on Gabapentin to conventional regimen.

This study has been conducted in a cross sectional manner. Clinical data was obtained from medical records of patients archived at the office of a physician with well established expertise in pediatric neurology. Patients with seizures not being responsive to two or three conventional AEDs were included in the study [11]. Cases of nonepileptic seizures and misdiagnosis were excluded. Regimens were categorized into eight groups. The first group consisting in conventional AEDs was considered as comparator and the effectiveness of other groups were compared with it. Each of the new antiepileptic drugs along with the regimen composed of conventional AEDs was incorporated into a model resulting in seven decision trees (sample shown in Figure 1).

**Figure 2:**
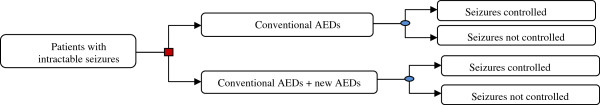
Decision tree of adding-on new AEDs to conventional regimens.

An additional model comparing regimens composed of conventional AEDs and any of the seven new AEDs with the same assumptions mentioned above was set up in order to evaluate the cost-effectiveness of adding-on new AEDs in general (Figure 2).

Regarding the fact that either the inability of a regimen in controlling seizures or the inappropriate safety profile are the main reasons of switching the regimen by the clinical specialist, thus remaining on a regimen has been viewed as an acceptable indicator of effectiveness and safety for regimens. Receiving approval by the clinician, remaining on a regimen for three months and more was regarded as effectiveness endpoint (desired consequence).

The proportion of seizure controlled patients to all the patients incorporated into the model was assumed as the effectiveness of the related regimen. The effectiveness of regimens was calculated for both arms within each model. Next the effectiveness of add-on regimens was subtracted from the comparator’s regimen effectiveness.

(1) $$\Delta E=E_{\text{add}-\text{on}}-E_{\text{comparator}}$$

Cost data was primarily obtained from the referral pharmacy of Tehran (Sizdah-e-Aban Pharmacy). Given the perspective of our study has been that of the patients, latest sales price were collected and split in two sets. The first set made up of the maximum prices consisting of brand name drugs that at most cases are imported and the second set comprised of the minimum prices which belong to the generic drugs. As availability of drugs has been the only determinant factor in purchasing drugs, an average of these prices was computed. Considering the variety of the dosage forms, standardizing the daily dose of each drug was necessary in order to compare the cost of each regimen. This was managed by calculating the prescribed daily dose (PDD) of each drug as an average of all the existing doses reported in the records.

(2) $$\text{average}\;\text{PDD}=\frac{\left(\text{dose}\;1\times \text{frequency}\;\text{of}\;\text{dose}\;1)+(\text{dose}\;2\times \text{frequency}\;\text{of}\;\text{dose}\;2\right)}{\text{frequency}\;\text{of}\;\text{dose}\;1+\text{frequency}\;\text{of}\;\text{dose}\;2}$$

For example if Valproate was prescribed as 900 mg/day in two records and 600 mg/day in one record the weighted average of PDD would equal 800 mg/day. PDD of each AED was subsequently multiplied by the price of 1 mg of each drug -obtained by dividing the average price by the potency of the dosage form- as well as 365 days of year for further comparison with the GDP (Gross Domestic Product) per capita, resulting in the cost of each regimen. The cost of the comparator group (conventional AEDs) has not been calculated due to being mentioned in both arms of the decision trees. In other words the difference of costs of regimens (ΔC) was simplified as followed:

(3) $$\Delta C=C_{\text{Conventional}+\text{new}\;\text{AED}}-C_{\text{conventional}}=C_{\text{new}\;\text{AED}}\text{.}$$

In order to compare the cost effectiveness of add-on regimens the incremental cost-effectiveness ratio (ICER) was calculated as:

(4) $$\text{ICER}=\frac{\Delta C}{\Delta E}$$

[12].

The final step was comparing the ICER of each new AED as add-on therapy with the GDP per capita of Iran, as a measure for assessing the cost-effectiveness of different regimens.

It should be noted that all costs are reported as Rials (Rls) (1 United States of America’ Dollar (USD) ~ 10,000 Rls; 1 Euro ~ 15,000 Rls) in year 2010.

Sensitivity analysis was performed at three cost levels (medium, average and maximum) and the model was rerun with an effectiveness endpoint of six months.

## Results

57 patients were approved with intractable seizures giving access to 284 records of regimens. The distribution of patients has been depicted (Table 1).

**Table 1:** Distribution of patients with intractable seizures

		**No.**	**% of total**
Sex	Male	32	56.14
	Female	25	43.86
Age	0–3 years	13	22.8
	3–6 years	20	35.09
	6–13 years	23	4.35
	13–19 years	1	1.75
Epilepsy type	Idiopathic	13	21.6
	Symptomatic	44	77.6
	Partial	27	57.36
	Generalized	9	15.78
	Mixed	21	36.84

### ICERs regarding at least 3 months of maintenance

Table 2 shows the incremental cost-effectiveness ratio of adding-on new AEDs in three months with average prices, ranges from negative values for Gabapentin, Levetiracetam and Zonisamide indicating them as dominated regimens to positive values for Vigabatrin (ICER ~ 43.7 mil Rls/consq), Topiramate (ICER ~ 21 mil Rls/consq), Oxcarbazepine (ICER ~ 7.7 mil Rls/consq) and Lamotrigine (ICER ~ 6.3 mil Rls/consq).

**Table 2:** Cost-effectiveness comparison of newer AEDs in three months at average prices

Regimen	**ΔE**	**ΔC**	**ICER (Rials per consequence)**
conventional AEDs+ Gabapentin	−0.52	1525051	Dominated
conventional AEDs+ Lamotrigine	0.13	826145	6354964
conventional AEDs+ Levetiracetam	−0.52	1732229	Dominated
conventional AEDs+ Oxcarbazepine	0.15	1149750	7665000
conventional AEDs+ Topiramate	0.36	7553771	20982698
conventional AEDs+ Vigabatrin	0.15	6560510	43736733
conventional AEDs+ Zonisamide	−0.52	1445400	Dominated

Note: the regimen solely composed of old AEDs is assumed as the comparator regimen.

### ICERs regarding the overall effect of adding-on new AEDs

The incremental cost-effectiveness ratio of adding any of the new AEDs shows a positive value of 37 million Rials per a desired consequence. Changing the effectiveness endpoint from three to six months raises the ICER value to 42.4 mil Rls/consq (Table 3).

**Table 3:** Cost-effectiveness comparison of adding-on new AEDs in three and six months at average prices

**Endpoint**	**ΔE**	**ΔC (Rials)**	**ICER**
3 months	0.08	2970408	37130101
6 months	0.07	2970408	42434402

### Comparison with GDP per capita

**Figure 3:**
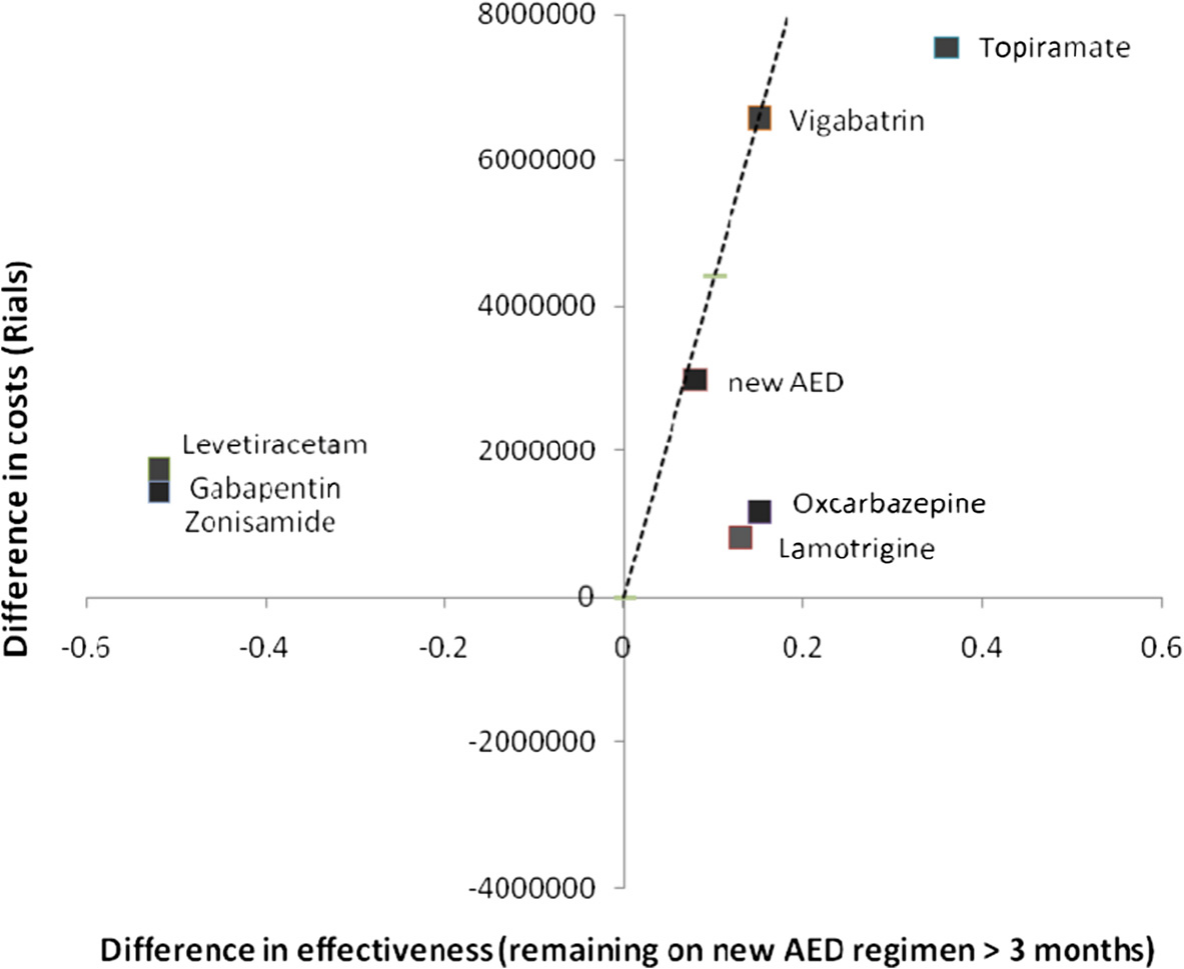
Cost-effectiveness analysis; Comparison of the ICER of adding-on new AEDs with the GDP per capita level.

As depicted in Figure 3 all new AEDs excluding Levetiracetam, Gabapentin and Zonisamide fall under the GDP per capita curve. It is noteworthy that Lamotrigine, Oxcarbazepine and Topiramate keep a distance from the curve while Vigabatrin and new AEDs point stand close to the line.

### Sensitivity analysis

**Figure 4:**
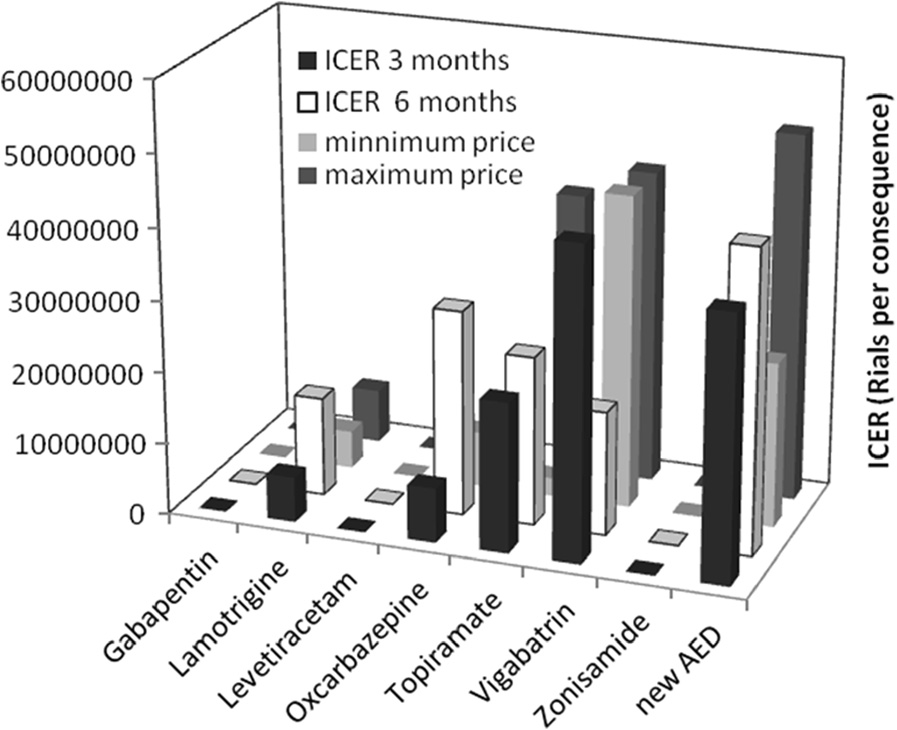
ICER sensitivity analysis at different price levels and different clinical endpoints.

Taking the case of two other price levels, can cause changes in the ICER values (Figure 4). Increasing the prices ends in falling the ICER of the new AEDs group out of the very cost-effectiveness area (> GDP per capita). Decreasing the prices, results in the enhancement of cost-effectiveness of Topiramate comparing to the GDP per capita level, followed by improvement of the cost-effectiveness of the new AEDs.

By the limit time of six months, Gabapentin, Levetiracetam and Zonisamide still show negative values. Oxcarbazepine (ICER ~ 28.7 mil Rls/consq) turns out to exhibit dramatic changes in the cost-effectiveness due to striking decrease in effectiveness, followed by Topiramate (ICER ~ 23.6 mil Rls/consq). The increase for Lamotrigine (ICER ~ 13.8 mil Rls/consq) has been less considerable. Opposite to the previous regimens Vigabatrin (ICER ~ 17.3 mil Rls/consq) has shown a substantial decrease.

## Discussion

The results confirm that adding-on new AEDs to conventional regimens in controlling intractable seizures in children is cost-effective comparing to regimens composed entirely of conventional AEDs. However choosing certain AEDs added to the conventional regimen especially in long term requires extra consideration. Given the general advantages of monotherapy against polytherapy, physicians’ preference could be driven towards monotherapy by cost-effective regimens including newer AEDs such as Oxcarbazepine, Lamotrigine and Topiramate.

Since the value that Iranian society recognize for health outcomes is unknown, interpreting the results express complicacy [13]. WHO guidelines recommend comparing the cost-effective ratio (CER) with the GDP per capita of the country [14]. The GDP per capita of Iran reported by the International monetary fund (2010) has been US$ 4400 [15] (~ 44 mil Rls). It should be noted that changes in any factor that modify the calculated costs such as exchange rates, prices and etc. can result in a different cost-effectiveness comparison. There lays a vast difference between these amounts and the ICER value of most regimens, allowing the prescription of the newer AEDs in large numbers. In spite of this, by considering the six months of retention as the desirable consequence, the ICER values of Oxcarbazepine at three price levels and Topiramate at the maximum price exceed the GDP per capita levels requiring more observation on their prescription. This is due to noticeable decrease in the efficacy of regimen.

If a specific regimen is causative of unacceptable side effects or is not efficient, the patient would have been quickly switched to an alternative regimen. Therefore retention rates of different regimens are appropriate indices for effectiveness. In addition actual clinical data used in this study instead of receiving abstract information by clinicians, improves realization of the results. While the study has much strength, cost and medical databanks not being available accounts for limitations through the study.

Collecting clinical data from a referral but single center can be responsible for undesirable bias in the results. Indirect costs such as costs pertaining to side effects and clinical visits were not included in this economic evaluation and different types of epilepsy were not separated due to the low number of patients.

Our results are quite different from Frew’s study that has demonstrated the newer and older antiepileptic drugs different in cost terms while equal in efficacy term [16]. This conflict arises from the different target group. Similarly, Knoester’s and Connek’s studies confirmed the use of older AEDs for patients newly diagnosed epilepsy while not included refractory patients [2,17]. Some studies such as Boon et al. have shown the favorableness of surgery in refractory patients compared with conservative treatment [18]. In general due to methodological issues comparing different studies is not always possible [19] and using standardized approach is suggested for further researches.

## Conclusion

Adding-on new antiepileptics to conventional regimens is cost-effective and justified considering the GDP per capita. Among the new adding-on AEDs prescribed in Iran, Lamotrigine shows the best results in terms of cost-effectiveness in treating children with intractable seizures. As the same, Oxcarbazepine and Topiramate fall under the GDP per capita level, while Vigabatrin stands close to the standard. However, other adding-on medications; Gabapentin, Levetiracetam and Zonisamide; in treating the target population appears not to be cost-effective due to less effectiveness compared with older AEDs.

## Competing interests

The authors declare that they have no competing interests.

## Authors’ contribution

ZG conceived and implemented the strategy, collected clinical and cost data, analyzed data and drafted paper, AK conceived the strategy of study and supervised the project, SN gave consultation on designing the study, conceived the strategy of study, revised the article and supervised the project, GZ gave consultation on designing the study and provided clinical data, AA gave consultation on designing the study and building the model. All authors read and approved the final manuscript.
